# Prognostic role of preoperative lymphocyte/C-reactive protein associated with upper gastrointestinal cancer: a meta-analysis

**DOI:** 10.3389/fonc.2023.1181649

**Published:** 2023-10-02

**Authors:** Yongjuan Ye, Guozhi Wu, Hao Yuan, Ya Zheng, Yuping Wang, Qinghong Guo

**Affiliations:** ^1^ The First Clinical Medical College, Lanzhou University, Lanzhou, China; ^2^ Department of Gastroenterology, The First Hospital of Lanzhou University, Lanzhou, China; ^3^ Key Laboratory for Gastrointestinal Diseases of Gansu Province, The First Hospital of Lanzhou University, Lanzhou, China

**Keywords:** esophageal cancer, gastric cancer, esophagogastric junction cancer, lymphocyte/C-reactive protein ratio, prognosis, meta-analysis

## Abstract

**Purpose:**

The lymphocyte/C-reactive protein (LCR) is a novel immunoinflammatory score and prognostic marker, but the relationship between lymphocyte/C-reactive proteins and clinical outcomes in patients with upper gastrointestinal cancers remains controversial. This study aimed to evaluate the relationship between LCR and the prognosis of upper gastrointestinal cancer by systematic evaluation and meta-analysis.

**Methods:**

We systematically searched PubMed, EMBASE, Cochrane, and Web of Science databases to obtain related studies on the relationship between LCR and esophageal cancer (EC), gastric cancer (GC), and esophagogastric junction cancers (EGJ), and used hazard ratio (HR), 95% confidence interval (95%CI) to evaluate the prognostic value of LCR. Outcome measures included overall survival (OS) and disease-free survival (DFS).

**Results:**

Eight retrospective cohort studies with 2838 patients were included. Meta-analysis showed that patients with low LCR cancers had poor overall survival OS and disease-free survival DFS (HR=2.18, 95%CI=1.87-2.55; HR=1.88, 95%CI=1.56-2.26). Subgroup analysis based on cancer type, treatment modality, gender, T stage, TNM stage, country, and LCR threshold showed that lower LCR levels were all associated with worse OS and DFS (P<0.05).

**Conclusion:**

The LCR can be used as a prognostic marker for patients with upper gastrointestinal cancers, and patients with a lower LCR may have a poor prognosis. Due to the limited number of studies included and mostly retrospective studies, the above findings require validation by more high-quality studies.

**Systematic Review Registration:**

https://www.crd.york.ac.uk, identifier CRD42023392433.

## 1 Introduction

Upper gastrointestinal cancer is one of the leading causes of cancer death worldwide. In 2020, there were more than 1.3 million upper gastrointestinal cancer-related deaths worldwide (1), among which GC ranked third (7.7%) and EC ranked sixth (5.5%). Moreover, with the aging of the population, the incidence of the upper digestive tract will continue to be high over the next decade. It is estimated that the incidence of GC in Asia can increase to about 20 people/100,000 (2), the global EC patients are expected to increase by 63.5% in 2040, and the number of deaths may increase by about 68% (3). Although advances in surgical techniques and drugs contribute to reducing the short-and long-term postoperative outcome risk, the prognosis of patients with upper gastrointestinal cancer remains poor. Therefore, it is crucial to determine the prognosis of patients with upper gastrointestinal cancer.

Currently, the TNM stage is the clearest prognostic indicator for cancer patients, but still inadequate (4). Several studies have shown that the prognosis of cancer patients depends on tumor and patient-related factors, where patient-related factors include inflammation, nutrition, and immune status. Cancer-associated inflammation is considered the seventh critical component of cancer (5, 6). Furthermore, it is well known that inflammation is closely related to cancer development, including carcinogenesis and tumor progression (e.g., invasion, migration, and metastasis) (7). There are some inflammatory markers like neutrophil-lymphocyte ratio (NLR) (8), platelet-lymphocyte ratio (PLR) (9), lymphocyte-monocyte ratio (LMR) (10), C-reactive protein/albumin (CAR) (11), Glasgow prognosis score (GPS), systemic inflammation score (SIS) (12), and prognostic nutrition index (PNI) etc. (13)

Lymphocytes can enhance immunosurveillance to suppress tumor development, so the increased number of lymphocytes indicates that the body’s effect on tumor suppression enhances (14). C-reactive protein (CRP) can increase rapidly in the case of inflammation, infection, and injury, activate complement and enhance the phagocytosis of phagocytes to eliminate the pathogenic microorganism (15). As an inflammatory marker, lymphocytes have high specificity but low sensitivity, CRP has low specificity but high sensitivity, and LCR may better reflect the inflammatory situation (16). LCR has been reported to be associated with prognosis in colorectal cancer (17), hepatocellular carcinoma (18), breast cancer (16), and lung cancer (19). This study aimed to evaluate the relationship between LCR and the prognosis of upper gastrointestinal cancer by systematic evaluation and meta-analysis.

## 2 Data and methods

### 2.1 Search strategy

Yongjuan Ye and Guozhi Wu searched the databases of PubMed, EMBASE, Cochrane, and Web of Science. They collected published cohort studies in July 2023 on the prognosis relationship of LCR, EC, and GC. The search strategy was conducted using a combination of subject terms and free words and a search of the references of the included articles. Search terms include Esophageal Neoplasms, Esophagus Cancer, Cancer of the Esophagus, Stomach Neoplasms, Gastric Cancer, Lymphocyte-to-C-reactive protein ratio, Lymphocyte to C reactive protein ratio, lymphocyte/C reactive protein, lymphocyte c-reactive protein ratio, LCR etc. This study was registered on the PROSPERO website (registration number: CRD42023392433). https://www.crd.york.ac.uk. The inclusion and exclusion criteria were as follows:

Inclusion criteria: (1) Study type: cohort study; (2) Study population: literature on published studies exploring the relationship between LCR and EC and GC; (3) Prognostic indicators: OS, DFS, recurrence-free survival (RFS).

Exclusion criteria: (1) Duplicate literature; (2) Article types are review, systematic evaluation, meetings, and comments; (3) The full text of the literature is not available; (4) Literature with inconsistent outcome indicators or insufficient data.

**Figure 1:**
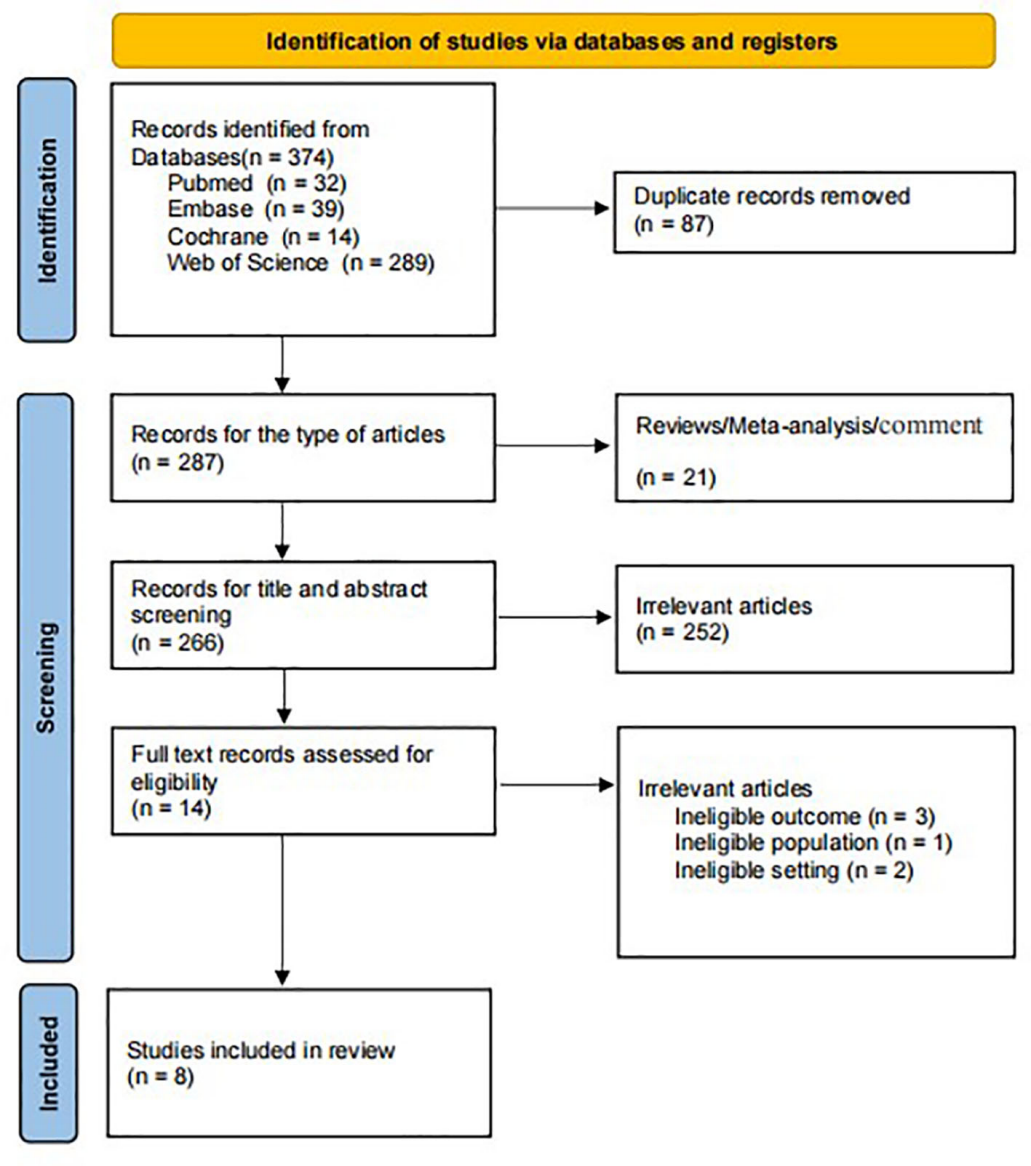
Flow chart for study identification and inclusion.

Yongjuan Ye and Guozhi Wu performed literature screening and data extraction, which screened the literature independently and extracted data diseases for cross-checking. They resolved objections by the third investigator—after reading the title and abstract, excluding irrelevant literature, and further reading the full text to determine inclusion. After a total of 374 studies excluding 87 duplicate articles, 21 reviews and meta-analysis, 251 unrelated articles, and seven inconsistent outcome measures, eight articles were published in 2020-2023 (**Figure 1**) (20–27). Data extraction included: first author, year of publication, cancer type, study duration, country, sample size, gender, treatment method, LCR threshold, threshold determination method, and outcome measures of interest. Literature quality was assessed using the Newcastle-Ottawa Scale (NOS), and studies with a score of 6 or higher were defined as high-quality studies.

### 2.2 Statistical processing

The HR and its 95% CI were used to evaluate the relationship between LCR and the prognosis in patients with upper gastrointestinal cancer. Q test was used to assess the heterogeneity of inclusion tests, I^2^<50% or P>0.1 was considered insignificant, and fixed effects model for analysis (28); sensitivity analysis was performed by removing one study or several studies at a time to verify the robustness of OS and DFS results. Funnel plot, Begg’s test, and Egger’s test were used to evaluate publication bias with a P-value of 0.05. All statistical analyses were performed using versions RevMan 5.4 and STATA 17.0.

## 3 Results

### 3.1 Essential characteristics of the included studies

The characteristics of the included studies are summarized in **Table 1**. Eight studies were included, all retrospective, including 2838 patients. Among the included articles, 8 investigated the prognostic role of LCR on OS in EC, GC, and esophagogastric junction cancers, and 6 investigated the prognostic role of LCR in upper gastrointestinal cancer on DFS. Seven studies were from Japan, and one study was from China. NOS score of 6 and above and defined as high-quality studies.

**Table 1:** Characteristics of included studies.

Included studies	Country	Type	Research period	Gender	stage	Treatment	LCR cutoff	LCR determined way	Outcome	NOS
2020 Cheng	China	GC	2013-2019	196:411	I-III	Surgery + post-chemotherapy	6300	ROC analysis	OS、DFS	6
2020 Okugawa	Japan	GC	2001-2011	387:164	I-IV	Surgery + post-chemotherapy	OS:8350 DFS:8350	ROC analysis	OS、DFS	8
2021 Takeuchi	Japan	EC	2000-2019	421:74	I-IV	Surgery + pre-chemotherapy	19000	ROC analysis	OS、DFS	7
2021 Yamamoto	Japan	EC	2002-2017	128:25	I-IV	Surgery + pre-chemotherapy	7842	ROC analysis	OS、DFS	8
2022 Aoyama	Japan	GC	2013-2017	318:162	I-III	Surgery + pre-chemotherapy	7000	ROC analysis	OS、DFS	8
2022 Sugawara	Japan	EC	2006-2017	308:52	——	Surgery + post-chemotherapy	6029.9	ROC analysis	OS、DFS	7
2022 Tsujiura	Japan	EGJ	2002-2020	85:18	I-IV	Surgery + pre/post-chemotherapy	4610	ROC analysis	OS、DFS	7
2023 Aoyama	Japan	EC	2008-2018	77:12	I-IV	Surgery + post-chemotherapy	12177	ROC analysis	OS、DFS	7

GC, gastric cancer; EC, esophageal cancer; EGJ, esophago-gastric junction cancer.

### 3.2 Meta-analysis results

#### 3.2.1 The relationship between LCR and OS

**Figure 2:**
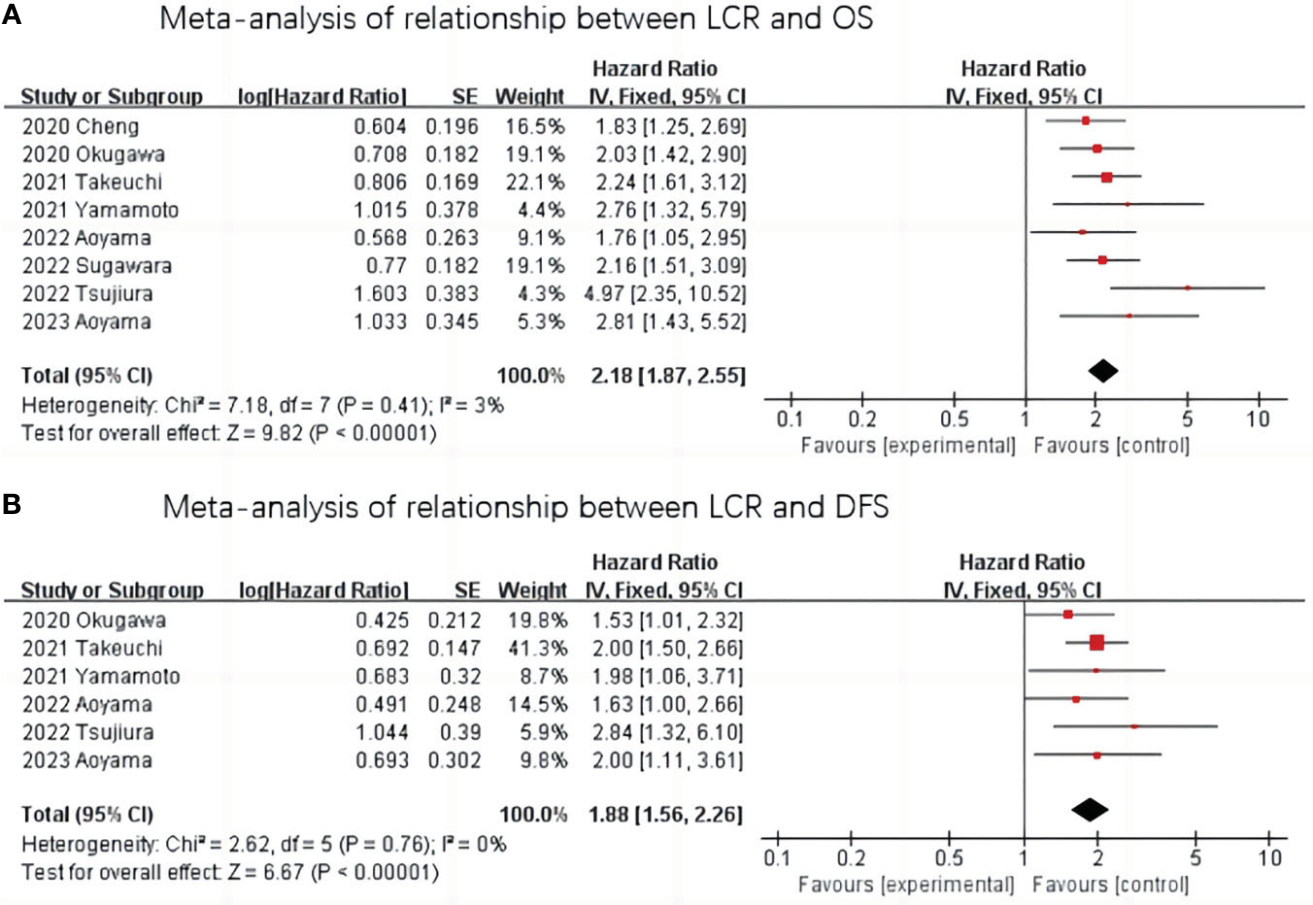
Meta-analysis of the association between LCR and prognosis (OS and DFS) in patients with upper gastrointestinal cancer. **(A)** Meta-analysis of relationship between LCR and OS. **(B)** Meta-analysis of relationship between LCR and DFS.

The relationship between LCR and OS was reported in eight studies with no significant heterogeneity between studies (I^2^ = 3%, P=0.41), so a fixed-effect model was used. The Meta-analysis showed that patients with lower LCR levels had significantly worse OS (HR=2.18, 95%CI=1.87-2.55, P <0.001)—**Figure 2A**.

#### 3.2.2 Relationship between LCR and DFS

The relationship between LCR and DFS was reported in six studies, and there was no heterogeneity between the studies (I^2^ = 0%, P=0.76), so a fixed-effect model was used. The results of the Meta-analysis showed that patients with lower LCR levels had significantly worse DFS (HR=1.88,95%CI=1.56-2.26, P< 0.001) —**Figure 2B**.

### 3.3 Subgroup analysis

**Figure 3:**
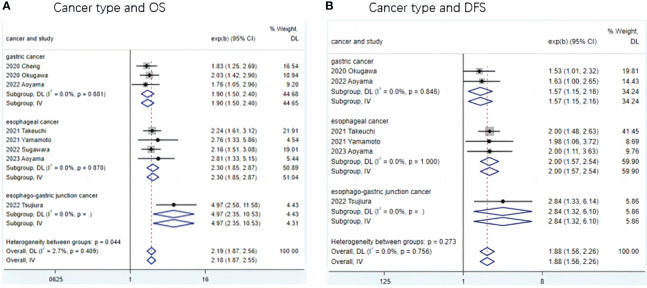
Subgroup analysis of the association between LCR and prognosis (OS and DFS) in patients with different type upper gastrointestinal cancer. **(A)** Meta-analysis of relationship between cancer type and OS **(B)** Meta-analysis of relationship between cancer type and DFS.

To further investigate the prognostic value of LCR in patients with upper gastrointestinal cancers, this study performed a subgroup analysis in terms of cancer type, country, LCR threshold, treatment modality, and TNM stage. Low LCR was associated with poorer OS. The heterogeneity of different cancer types was weak (I^2^ = 0, P<0.1), meaning that cancers in different parts of the upper digestive tract had less influence on the meta-analysis results. Based on the above analysis, patients with lower LCR levels had significantly worse OS and DFS (**Figure 3**). Moreover, our analysis of the relationship between LCR and gender and T stage showed that patients in the low LCR group had worse T stage (OR=0.68, 95%CI=0.59-0.78, P<0.001), and men were more likely to have low LCR (OR=1.15, 95%CI=1.00-1.33, P=0.05) (**Table 2**).

**Table 2a:** Subgroup analysis of relationship between LCR and OS.

Characteristic	Number of studies	Sample size	Model	HR(95%CI)	P	Heterogeneity	
						I^2^	P
type							
EC	4	1097	FED	2.30 (1.85,2.87)	<0.001	0	0.87
GC	3	1638	FED	1.90 (1.50,240)	<0.001	0	0.88
EGJ	1	103	FED	4.97 (2.35,10.52)	<0.001		
country							
China	1	607	FED	1.83 (1.25,2.69)	<0.001		
Japan	7	2231	FED	2.26 (1.91,2.68)	<0.001	3	0.4
LCR cutoff							
≤7000	4	1550	RED	2.21 (1.58,3.08)	<0.001	50	0.11
>7000	4	1288	FED	2.25 (1.81,2.80)	<0.001	0	0.79
treatment							
Surgery + pre-chemotherapy	3	1128	FED	2.16 (1.67,2.81)	<0.001	0	0.59
Surgery + pre/post-chemotherapy	1	103	FED	4.97 (2.35,10.53)	<0.001	0	
Surgery + post-chemotherapy	4	1607	FED	2.07 (1.69,2.53)	<0.001	0	0.74
TNM stage							
I-III	2	1087	FED	1.80 (1.33,2.45)	<0.001	0	0.91
I-IV	5	1391	FED	2.39 (1.94,2.95)	<0.001	19	0.29

FED, fixed effect model; RED, random effect model; HR, The high LCR group is the compared group.

**Table 2b:** Relations of LCR with upper gastrointestinal cancer patients.

Characteristic	Number of studies	Sample size	Model	OR (95%CI)	P	Heterogeneity	
						I^2^	P
sex(male/female)	3	1582	FED	1.15 (1.00,1.33)	0.05	0	0.68
T stage(T1/T2-3)	3	1412	FED	0.68 (0.59,0.78)	<0.001	35	0.22

## 4 Publication bias

**Figure 4:**
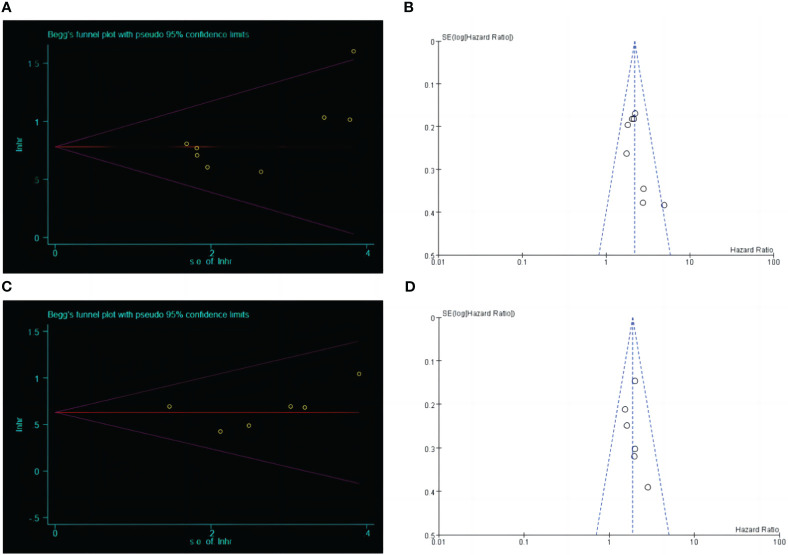
Begg's test and funnel plot analysis between LCR and prognosis (OS and DFS) in patients with upper gastrointestinal cancer. **(A)** Begg's test between LCR and OS. **(B)** Funnel plot analysis between LCR and OS. **(C)** Begg's test between LCR and DFS. **(D)** Funnel plot analysis between LCR and DFS.

The relationship between LCR and OS was performed by Begg’s test (Z=0.87, P=0.386), Egger’s test (t=2.09, P=0.081), funnel Fig, publication bias between LCR and DFS was performed by Begg’s test (Z=0.75, P=0.452), Egger’s test (t=0.53, P=0.627), and funnel Fig. The results indicated that the included literature had a low possibility of publication bias (**Figure 4**).

### 4.1 Sensitivity analysis

**Figure 5:**
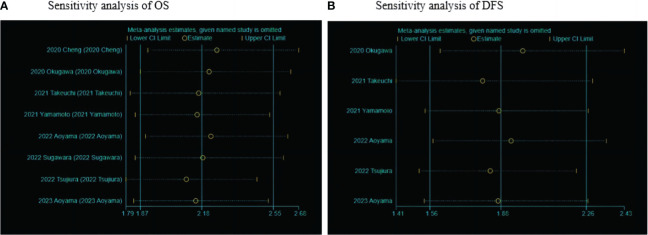
Sensitivity analysis of LCR and prognosis (OS and DFS) in patients with upper gastrointestinal cancers. **(A)** Sensitivity analysis between LCR and OS. **(B)** Sensitivity analysis between LCR and DFS.

Sensitivity analysis was performed by excluding individual studies, showing a stable meta-analysis of the relationship between LCR and OS and a meta-analysis of the relationship between LCR and DFS (**Figure 5**).

## 5 Discussions

Surgery can lead to the release of proinflammatory factors, especially IL-6, then induce lymphocytes and increased CRP secretion, so postoperative LCR may be the body to surgical stress response (29), and a study (30) reported that the systemic inflammatory response level to preoperative level requires 21-90 days, so this paper is dedicated to evaluating the predictive value of preoperative LCR for postoperative overall survival and disease-free survival of upper gastrointestinal cancer. An increasing number of studies have shown that low preoperative LCR is associated with poor prognosis in patients with upper gastrointestinal cancer. In eight clinical studies, 2838 patients with upper gastrointestinal cancers were meta-analyzed to assess the prognostic impact of LCR. The results showed a correlation between LCR levels and shorter OS and DFS in patients with upper gastrointestinal cancers.

Characteristics of tumor-associated inflammation include the infiltration of inflammatory cells, the production of inflammatory factors in the tumor tissue, tissue remodeling, tissue repair, and angiogenic (6). In the tumor microenvironment, the presence of lymphocytes in the infiltrating margin and cancer nest is associated with the prognosis of cancer, and lymphocytes inhibit the proliferation and metastasis of tumor cells from promoting immune response (31); CRP is the most commonly used marker to evaluate the degree of inflammation (32), secreted by liver oocytes, rapidly in the acute stage of inflammation, mild increase in chronic inflammation or cancer, CRP interacts with inflammatory cells and stromal cells in the tumor microenvironment, reflecting the proliferation and metastasis of tumor cells, and is a marker of cancer development risk and prognosis (31).

Of the eight studies included in this paper, four studies (20, 22, 25, 26) reported that low LCR was a predictor of surgical site infection after surgery, which was also confirmed in a Turkish study (33) and Yildirim (34) et al. found that LCR had the highest accuracy when predicting complications on the fifth postoperative day after surgery. Okugawa et al. (20) reported that low LCR was associated with GC liver metastasis and peritoneal metastasis, but the results were not uniform in age, sex, pathological type, lymph node metastasis, and vascular infiltration. In eight studies, only Cheng (25) and Takeuchi (21) et al. reported no significant correlation between low LCR and tumor location, while the other studies did not mention whether the correlation; only Tsujiura et al. (24) reported no significant correlation between low LCR and BMI and tumor differentiation. The other studies did not mention whether the correlation, in four studies (20, 21, 25, 26) reported no significant correlation between low LCR and tumor differentiation, while in the correlation analysis between colorectal cancer and LCR, some studies (35) reported low differentiation in low LCR group. In addition, two studies (25, 26) reported that low LCR was significantly associated with GC size. Cheng et al. (25) found that LCR had the highest predictive OS of GC in CAR, LMR, NLR, PLR and LCR (AUC: 0.695) (25). The three studies included (20, 22, 23) and one Turkish study (33) reported that LCR was significantly associated with the TNM stage in GC patients. Miyatani et al. (32) found that combining preoperative and postoperative LCR had a poor prognosis for patients with GC at low levels (5-year survival rate 52.0%). Xu et al. (36) studied 262 patients with radical GC surgery and found that CLR was closely associated with the lymphovascular invasion status in GC patients (HR:1.73,95%CI:1.04-2.87, P=0.036). Due to the few studies included in this paper, further studies are still needed in the future to clarify the correlation between LCR and each clinicopathological characteristics. In conclusion, LCR has the highest prognostic value in various inflammatory indicators, and is also related to postoperative short-term prognosis, suggesting that we can not only predict a preoperative diagnosis of upper gastrointestinal cancer according to LCR, identify high-risk surgical patients, guide in treatment selection, perioperative immune regulation and nutritional support, can also predict the long-term prognosis, guidance in follow-up.

Although this meta-analysis indicates a poor prognosis for patients with upper gastrointestinal cancers with low preoperative LCR, but there are still some deficiencies to improve, the existing limitations are as follows: First, only eight studies were included in this article, 4 EC, 3 GC and 1 EGJ, a total of 2838 patients were included, there are certain limitations, the results need more high-quality literature to verify and supplement; Second, the included studies were all obtained from Asia, and seven are all from Japan, there are geographical differences, Studies from other regions are needed to verify and supplement the results of this paper; Third, the studies included in this paper were all retrospective cohort studies, there may be a selection bias, prospective, multicenter randomized controlled trial is required for validation; Fourth, in the studies included in this article, the treatment method is not uniform, which may affect the positive relationship between LCR and OS and DFS; Fifth, the inconsistent cut of LCR among the studies included herein this article may affect the results of the survival analysis; Sixth, the studies included in this paper are not uniform regarding the timing of lymphocyte and CRP collection, and whether the LCR is a dynamic indicator that affects the results is currently unknown.

## 6 Conclusion

Our meta-analysis showed that low LCR was strongly associated with survival outcomes in patients with upper gastrointestinal cancers. LCR is easily obtained from clinical test results, cheap, convenient and reproducible sampling, and can be widely used in predicting upper gastrointestinal cancer prognosis, but further studies are needed to validate the results of this paper.

## Data availability statement

The original contributions presented in the study are included in the article/**Supplementary Material**. Further inquiries can be directed to the corresponding author.

## Author contributions

YY conceived and designed the study. YY and GW selected the studies and collected and analyzed the data. YY, GW, and QG drafted the paper. All authors revised the draft paper. All authors contributed to the article and approved the submitted version.
