# Supplementary material for: Prognostic role of preoperative lymphocyte/C-reactive protein associated with upper gastrointestinal cancer: a meta-analysis
Source: Front Oncol. 2023 Oct 2;13:1181649. doi: 10.3389/fonc.2023.1181649 (PMC10578962; doi:10.3389/fonc.2023.1181649)
Supplement: Supplementary file 2 [file DataSheet_2.pdf]

NOS

study	selection			comparability			outcome			total
	REC	SNBC	AE	DO	SC	AF	AO	FU	AFU	
2020 Cheng	0	1	1	1	1	1	1	0	0	6
2020 Okugawa	1	1	1	1	1	0	1	1	1	8
2021 Takeuchi	1	1	1	1	1	1	1	0	0	7
2021 Yamamoto	1	1	1	1	1	1	1	1	0	8
2022 Aoyama	1	1	1	1	1	0	1	1	1	8
2022 Sugawar	1	1	1	1	1	0	0	1	1	7
2022 Tsujiura	1	1	1	1	1	1	1	0	0	7
2023 Aoyama	1	1	1	1	0	0	1	1	1	7

REC, representativeness of the exposed cohort; SNEC, selection of the non-exposed cohort; AE, ascertainment of exposure; DO, demonstration that the outcome of interest was not present at the start of the study; SC, study controls for age, sex; AF, study controls for any additional factors (chemoradiotherapy, curative resection); AO, assessment of outcome; FU, follow-up long enough (60 Months) for outcomes to occur; AFU, adequacy of follow-up of cohorts (all subjects followed up for 5 years were 1). ‘1’ means that the study is satisfied the criterion, and ‘0’ means that it did not.
